# Supplementary material for: Electronic structure with direct diagonalization on a D-wave quantum annealer
Source: Sci Rep. 2020 Nov 27;10:20753. doi: 10.1038/s41598-020-77315-4 (PMC7695747; doi:10.1038/s41598-020-77315-4)
Supplement: Supplementary file 1 — Supplementary Information. [file 41598_2020_77315_MOESM1_ESM.pdf]

Supplementary Information

Electronic structure with direct diagonalization on a D-Wave quantum annealer

Alexander Teplukhin, Brian K. Kendrick, and Sergei Tretiak

Theoretical Division (T-1, MS B221), Los Alamos National Laboratory, Los Alamos, New Mexico 87545, USA

Pavel A. Dub

Chemistry Division (C-IIAC, MS K558), Los Alamos National Laboratory, Los Alamos, New Mexico 87545, USA

(Dated: November 12, 2020)

bkendric@lanl.gov and pdub@lanl.gov

TABLE S1. Electronic ground state energy of the H_2 molecule computed using the QAE and FCI matrices. The absolute energies are given in Hartrees (E_h), the differences are given in kcal/mol.

Basis	Mat. size	QUBO size	E_{ref} [a]	E_{cl} [b]	E_{hw} [c]	$E_{cl} - E_{hw}$	$E_{hw} - E_{ref}$
STO-3G	2x2	20	-1.13684890	-1.13684890	-1.13684890	0.000	0.000
3-21G	8x8	80	-1.14772560	-1.14772396	-1.14772157	-0.001	0.003
6-31G	8x8	80	-1.15152414	-1.15145953	-1.15151476	0.035	0.006
cc-PVDZ	22x22	220	-1.16356044	-1.16260821	-1.16226936	-0.213	0.810
def2-TZVP	36x36	360	-1.16822660	-1.16798881	-1.16795448	-0.022	0.171
6-311++G**	54x54	540	-1.16832827	-1.16808739	-1.16763178	-0.286	0.437
aug-cc-PVDZ	66x66	660	-1.16477801	-1.15912926	-1.15921477	0.054	3.491
6-311++G2d,2p	88x88	880	-1.17082208	-1.16702940	-1.16504499	-1.245	3.625
cc-PVTZ	136x136	1360	-1.17229056	-1.16757762	-1.16662200	-0.600	3.557
6-311++G2df,2pd	166x166	1660	-1.17230096	-1.16788170	-1.16792099	0.025	2.748
aug-cc-PVTZ	350x350	3500	-1.17258553	-1.16637818	-1.16637972	0.001	3.894
cc-PVQZ	552x552	5520	-1.17375026	-1.16599589	-1.16604277	0.029	4.836
def2-QZVP	552x552	5520	-1.17382320	-1.16645322	-1.16653460	0.051	4.574
aug-cc-PVQZ	1256x1256	12560	-1.17382181	-1.16357644	No data [d]	-	-

[a] Energy obtained using the reference diagonalization.
[b] Energy obtained using the QAE in the classical mode (Tabu search).
[c] Energy obtained using the QAE in the hardware mode (D-Wave 2000Q).
[d] The QAE failed in the hardware mode (see text).

TABLE S2. Absolute electronic ground state energies (E_h) and energy differences (kcal/mol).

Molecule	Method	Basis	Mat. size	QUBO size	E_{ref} [a]	E_{cl} [b]	E_{hw} [c]	$E_{cl} - E_{hw}$	$E_{hw} - E_{ref}$
H_2	FCI	STO-3G	2x2	20	-1.13684890	-1.13684890	-1.13684890	0.000	0.000
HF	FCI	STO-3G	18x18	180	-98.60174790	-98.60150630	-98.60140647	-0.063	0.214
H_2O	FCI	STO-3G	133x133	1330	-75.02039100	-75.01165123	-75.01178703	0.085	5.399
H_2O	CAS(8e,7o)SCF	cc-PVDZ	321x321	3210	-76.11470570	-76.10050279	-76.10314815	1.660	7.252
CH_2^{2+}	FCI	STO-3G	169x169	1690	-37.40440097	-37.39601005	-37.39647755	0.293	4.972
BeH_2	FCI	STO-3G	169x169	1690	-15.59474568	-15.59143293	-15.59095276	-0.301	2.380
H_3^+	FCI	cc-PVTZ	532x532	5320	-1.34149794	-1.33044775	-1.33033386	-0.071	7.005
BH_3	CAS(6e,6o)SCF	6-311++G**	208x208	2080	-26.44129518	-26.43066167	-26.42990861	-0.473	7.145
BH_3	FCI	STO-3G	1250x1250	12500	-26.12145752	-26.10470471	No data [d]	-	-

[a] Energy obtained using the reference diagonalization.

[b] Energy obtained using the QAE in the classical mode (Tabu search).

[c] Energy obtained using the QAE in the hardware mode (D-Wave 2000Q).

[d] The QAE failed in the hardware mode (see text).

TABLE S3. Absolute electronic excited state energies (E_h) and energy differences (kcal/mol) of the H_2O molecule computed using the FCI/STO-3G matrix.

State #	E_{ref} [a]	E_{cl} [b]	E_{hw} [c]	$E_{cl} - E_{hw}$	$E_{hw} - E_{ref}$
1	-75.02039100	-75.00825860	-75.01096336	1.697	5.916
2	-74.53743573	-74.52927677	-74.52949083	0.134	4.985
3	-74.43197396	-74.41290660	-74.40564845	-4.555	16.519
4	-74.31751275	-74.31150179	-74.30211833	-5.888	9.660
5	-74.07950770	-74.04154252	-74.03510825	-4.038	27.861

[a] Energy obtained using the reference diagonalization.

[b] Energy obtained using the QAE in the classical mode (Tabu search).

[c] Energy obtained using the QAE in the hardware mode (D-Wave 2000Q).

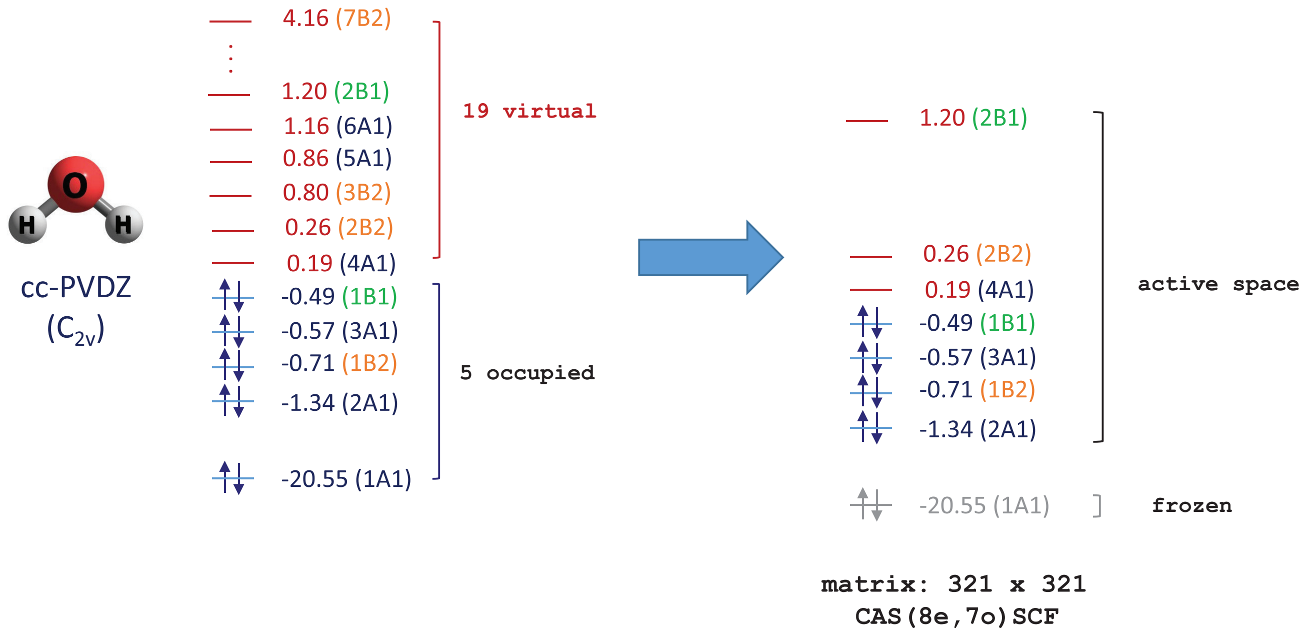

FIG. S1. Active space selection for the H_2O molecule. The 2 lowest-energy electrons out of 10 were frozen (core) and 7 orbitals were selected out of 24. The resulting CAS(8e,7o)SCF calculation was done using the cc-PVDZ basis set.

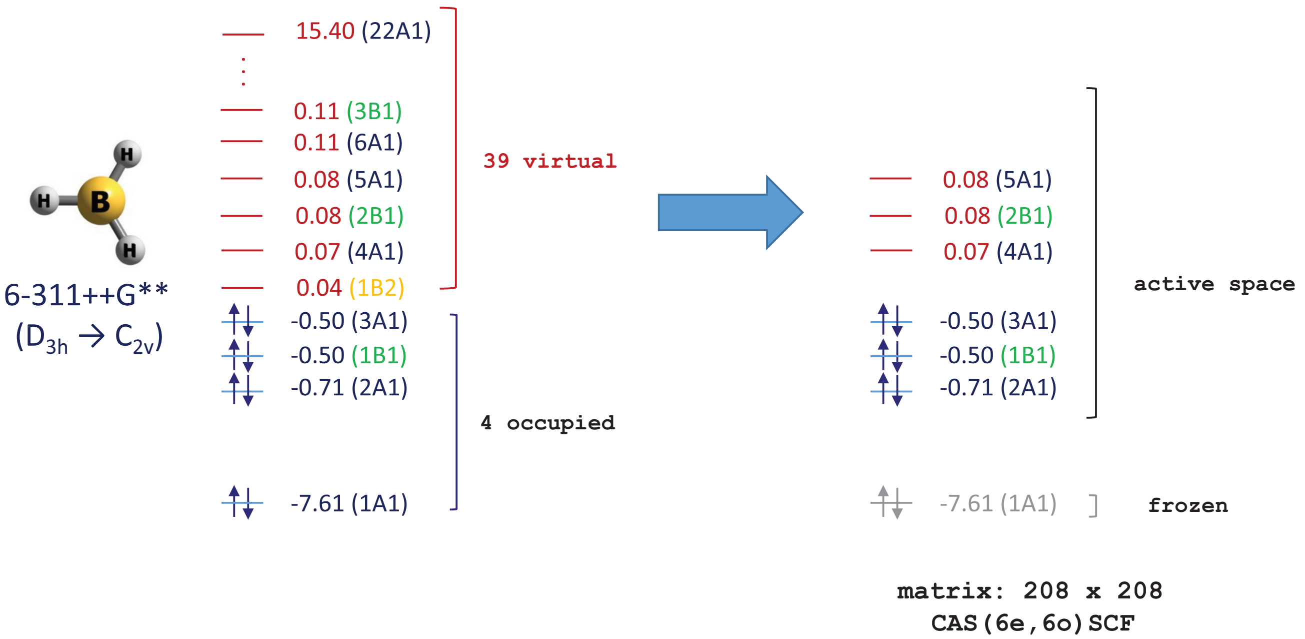

FIG. S2. Active space selection for the BH_3 molecule. The 2 lowest-energy electrons out of 8 were frozen (core) and 6 orbitals were selected out of 43. The resulting CAS(6e,6o)SCF calculation was done using the 6-311++G** basis set.
